# Making social sciences foundational to academic medicine

**DOI:** 10.1371/journal.pmed.1004649

**Published:** 2025-06-16

**Authors:** Sahar Sadjadi, Lisa Barkley, Rebecca Jordan-Young, Helena Hansen

**Affiliations:** 1 Department of Social Studies of Medicine, McGill University, Montreal, Quebec, Canada; 2 Department of Family Medicine, College of Medicine, Charles R. Drew University of Medicine & Science, Los Angeles, California, United States of America; 3 Department of Women’s, Gender and Sexuality Studies, Barnard College and Center for Science and Society, Columbia University, New York, New York, United States of America; 4 Department of Psychiatry, Semel Institute for Neuroscience and Human Behavior and Center for Social Medicine, University of California, Los Angeles, California, United States of America

## Abstract

In this Perspective, Sahar Sadjadi and colleagues discuss the imperative for incorporating social sciences into medical research, education and implementation.

## Teaser

Despite longstanding evidence of the enormous impact of socioeconomic conditions on population health, social sciences remain marginal to academic medicine. In this Perspective, we propose the integration of social sciences into the three pillars of academic medicine—education, research, and clinical practice—to build the theoretical and methodological foundations for identifying the mechanisms through which social and community factors shape health and disease, and to advance sustainable community engagement in the creation of medical knowledge.

The current state of United States (US) health and healthcare demands a rethinking of academic medicine. Despite spending the most on healthcare per capita in the world, the US has the worst health outcomes among high-income nations with the lowest life expectancy at birth and highest infant and maternal mortality rates [1]. Maternal mortality rate has increased in the last two decades with wide racial disparities [2]. As an entity with outsized influence on standards of care and medical knowledge, and a recipient of massive financial investment, academic medicine has a specific role to play in changing healthcare priorities. Social sciences should be integral to that role.

Solutions require moving beyond individual level healthcare to include addressing the socioeconomic conditions that have a greater impact on mortality, well-being, and quality of life [3]. Yet, US academic medicine reflects the disproportionate investment in the costly precision diagnostic tools, tertiary care at the end of life, biotechnology, and pharmaceuticals. These advances can come at the expense of population-based, comprehensive, and preventive care strategies that address Social Determinants of Health, including inequality and structural racism.

Leading academic medicine institutions, including the Association of American Medical Colleges andthe National Institutes of Health, have acknowledged the need for socially conscious perspectives and set goals for addressing social factors. We applaud these commitments, but they fall short of the changes in framework and investment that the problem requires.

The traditional knowledge base for translational medicine—which typically spans from basic science to technology to bedside—is inadequate to address the social drivers of poor health. Operationalizing the “Social” in Social Determinants of Health is not intuitive; moving beyond quantifying the size of health inequalities toward understanding the mechanisms by which community conditions, institutional practices and public policies influence human biology requires the methodological and conceptual tools of the social sciences. Moreover, social science-informed standards for community engagement in biomedical research could help to redress public distrust of biomedicine. Yet, social sciences such as sociology, anthropology and history, remain siloed from academic medicine—a major, and consequential, missed opportunity to improve population health.

This essay has emerged from a series of discussions over healthcare in the United States, a context that lacks a robust tradition of social medicine seen elsewhere, for example in Latin American countries [3]. At the same time, the US academic institutions and research infrastructure and funds have exerted enormous influence on shaping global patterns in medical research and education and the development of biomedical knowledge and technologies, rendering our invitation to further incorporate social sciences relevant beyond the United States. Current US withdrawal from longstanding international partnerships and global health initiatives has destructive consequences but also opens the space for new conversations about reorienting health research and healthcare investments toward a more socially conscious medicine on the other side of the crisis. As health practitioners and social scientists who work within medical institutions, with a role in training future physicians and allied health professionals, we offer this preliminary vision for the integration of social sciences into the three pillars of academic medicine: education, research, and clinical practice (see Table 1 for examples). Strengthening community engagement through each of these pillars is crucial for ethical and effective healthcare.

**Table 1:** Selected examples: integration of social sciences into medicine (click hyperlink for details).

Education	Research	Clinical Practice
Structural Competency Curriculum: Clinical Skills for Structural Intervention structuralcompetency.org *This set of competencies requires clinicians to address structural drivers of health through collaborations with community organizations, non-health agencies and policy makers*	Symbioses: A Biosocial Research Network A collaborative network of life scientists, social scientists and clinical researchers studying the mechanisms by which social environments shape human biology	Medical Legal Partnerships Collaborations of pro-bono lawyers or law students with clinicians to pursue patients’ cases legally when warranted (e.g., housing tenant rights, disability accommodations)
Walking Classroom: Clinical Trainees Building Relationships with Burdened Communities Clinical trainees and residents take tours of neighborhoods from which patients come, with local community organization guides	University of California Symposium on The Use of Race in Medicine Critical review of racial categories in medical research and clinical guidelines	Peer Navigators/Community Health Workers Evidence based employment of local community members and people with lived experience to liaison with patients as a part of the clinical care team
Community Leaders as Clinical Instructors, Teaching Neighborhoods	Robert Wood Johnson Foundation Culture of Health Initiative A health in all policies approach calling on all sectors (e.g., transportation, parks and rec) to promote health	Locating Clinical Care in Community Organizations Examples include Imani Breakthrough Project, locating addiction treatment in faith based organizations https://portal.ct.gov/dmhas/newsworthy/news-items/the-imani-breakthrough-project
From Histories of Segregated Cities to Urban Design for Integration and Health	National Academy of Medicine: Integrating Social Care	Clinician Advocacy for Health in all Policies
University of California PRIME Program: Addressing Social Determinants in Primary Care	Citizen Science by People with Lived Experience—example of Urban Survivor’s Union “Methadone Manifesto”	Collective Recovery An evidence-based approach to mental health recovery after disaster involving community-based mutual aid, the arts and greenspace programs

## Education

Accreditation standards already require that medical curricula address social, political, economic, and cultural factors that influence health. Yet most clinical faculty have no background in the social sciences, and in the absence of a theoretically and methodologically rigorous approach, well-intentioned initiatives unintentionally reinforce erroneous assumptions and ineffective interventions, for which social scientists provide crucial correctives.

For example, social scientists challenge the mischaracterization of race in medical education as a biological variable rather than a social category and illuminate historical roots of “race specific” data in clinical medicine algorithms as well as their perverse effects, that can amplify rather than ameliorate racial disparities in treatments and outcomes [4,5]. Similarly, correctives for cultural competency training can reproduce cultural stereotypes, suggesting that the beliefs and behaviors of patients themselves are responsible for unequal health outcomes. Dually trained physician-social scientists Hansen and Metzel introduced the alternative of “structural competency,” which focuses on how clinical practitioners can address the community conditions, institutional forces, and policies that drive health outcomes, in collaboration with community members, non-health sector agencies, and policy makers [6]. This insight and intervention stems from the theoretical and methodological repertoire of social sciences.

To tread the complex terrain at the interface of the biological and the social requires cultivating a deep understanding of both, as well as their interrelation. This endeavor can be mutually enriching for both social sciences and medicine [7–8]. For example, social scientific scholarship has retooled and enhanced one of the oldest and most important methods of clinicians, history taking, as seen in the movements to integrate narrative medicine [9], mini-ethnographies [10], and assessment of structural vulnerability into clinical training (see Table 1). Systematic observation, a bedrock of ethnographic method, is also crucial to medical care.

Achieving the goal of fully integrating social sciences in medical teaching practices will require:

1. Curriculum reform that prioritizes rigorous social science and community engagement;
2. Robust inclusion of social science and humanities scholars on academic medicine faculty and among those receiving medical research funding.

## Research

Social sciences generate advanced knowledge on complex health problems, yet their relational, systemic, and ecological methods differ from biomedical research methods—such as randomized control designs—which are currently favored by many funders. Cutting edge life science fields such as epigenetics, neuroplasticity, and the microbiome show how biological systems shape, and are shaped by, their environments, offering a new paradigm for translational science. Social sciences enable biosocial approaches, that regard biological and the social as mutually constitutive, in collaborations with life scientists and clinicians.

To include social sciences as basic sciences in the study of health and medicine would involve delineating paths from social science findings to the development of “social technologies,” i.e., community, institutional, and policy level health interventions that are necessary for the effectiveness of molecular technologies in improving health. Social science scholars are equipped to set standards for community engagement for creating medical knowledge. By practicing such engagement, they expand the expectation of community involvement to the basic sciences and clinical trials. This can help to redress a crisis of public trust in medical sciences and research participation, which is especially acute in communities of color [11].

To foster these approaches, clinical journals must publish social science and biosocial research and have social scientists on their editorial boards and among their reviewers to assess and elevate the standards of social scientific scholarship. In addition, health funders must have social scientists among their leaders and on grant review sections to evaluate proposals addressing relevant aims and methodologies. Requests for proposals can explicitly encourage structural analyses and require dissemination plans that elucidate systemic impact and engender research-community accountability. Such reforms could greatly shift the national priority setting toward social-structural health equity.

A model can be found in the Robert Wood Johnson Foundation’s Cultures of Health initiative that promotes health at systemic levels to operationalize health in all domains of policy making—in urban planning, transportation systems, and community cohesion in addition to healthcare delivery. This model shows that to transform biomedicine into a biosocial practice, social sciences must inform the development of community, institutional and policy interventions.

## Clinical practice

Unrealistic time constraints, fragmented and difficult-to-access systems of care, and payment models that reimburse medical procedures and specialty care at much higher rates than comprehensive primary care reinforce poor healthcare delivery. We need interprofessional clinical teams that partner with systems and organizations outside of healthcare to collaboratively apply the knowledge, skills, and resources to effectively address social dimensions of clinical care. Examples include medical-legal partnerships that address legal issues impacting health, housing assistance as healthcare, and food pharmacies for food insecurity. To achieve these goals, we need to shift funding priorities and the allocation of resources toward social factors that shape health and illness.

To understand the social conditions of the communities that they serve, clinical practitioners must also respond to community members’ knowledge of their own needs. Social sciences provide the tools to create community partnerships, including participatory program development that incorporates community member observations and analyses. Inadequate tools and resources for such partnerships can lead to unintended harms. Academic medicine should adopt the evidence base that comprehensive healthcare for all includes understanding the social context [11]. Examples of this include historically Black and Hispanic-serving medical schools in the United States that prioritize addressing social conditions of health and community engagement that foregrounds community knowledge and expertise.

Social scientists should provide the knowledge base for instructional teams—inclusive of community leaders, non-health sector leaders, community resources, and partnerships—to enhance shared decision-making and to close the gap between populations at the bottom and the top of the health gradient. To this end, social scientists must help clinical systems develop incentives and pay structures that align to include social indicators as quality care measures, such as value-based payment for performance on positive population health outcomes. Examples include innovation programs with the US Center for Medicare and Medicaid Innovation that provide financial incentives in health systems to provide social care and accountable care organization incentives to integrate social care into healthcare delivery [12]. They must also inform the design and dissemination of roadmaps to address socio-economic causes of poor health in clinical practice.

Academic medicine currently prioritizes individualized and fragmented solutions to complex health problems. Considering what we know about social determinants of health, the incorporation of social sciences into the pillars of academic medicine would only be consistent with its commitment to evidence-based practice. Social sciences are crucial to equipping future and current medical professionals to improve clinical care and population health.
